# Nickel oxide nanoparticles catalyst for enhancing green hydrogen production: effect of preparation conditions

**DOI:** 10.1186/s13065-025-01646-4

**Published:** 2025-10-27

**Authors:** Hatem A. Mahmoud, Aya Adel A. Ali, Tarek T. Ali, Bahaa M. Abu-Zied

**Affiliations:** 1 Chemistry Department, Faculty of Science, Sohag University, Sohag, 82524 Egypt; 2 Chemistry Department, Faculty of Science, Assiut University, Assiut, 71516 Egypt

**Keywords:** Nickel oxide, Hydrogen generation rate, Catalysis, Hydrothermal conditions, Green nickel oxide

## Abstract

This work focused on the synthesis of nickel oxide under varying hydrothermal conditions as a non-precious and efficient catalyst for NaBH_4_ hydrolysis to generate hydrogen. The structural, morphological, and textural features of the calcined samples were characterized using X-ray diffraction (XRD), Fourier-transform infrared spectroscopy (FT-IR), X-ray photoelectron spectroscopy (XPS), scanning electron microscopy (SEM), transmission electron microscopy (TEM), selected area electron diffraction (SAED), and N_2_-physisorption techniques. XPS analysis confirmed the presence of Ni^2+^ and Ni^3+^ species, with varying Ni^3+^/Ni^2+^ ratios across the samples. The NH-200 samples showed the highest hydrogen generation rate, approximately 1290 at 323 K, attributed to the enhanced redox behavior of Ni^3+^ ions. N_2_-adsorption results revealed mesoporous structures with distinct surface areas and pore characteristics. The catalytic performance was evaluated at 35–50 °C, showing enhanced activity with increasing temperature and NaBH_4_ concentration (up to 4.5 wt.%). Activation energies were found to be below 59 kJ mol^−1^. The catalyst maintained good stability over five consecutive cycles with only a slight performance decline. These findings confirm that NiO is a cost-effective, active, and reusable catalyst for hydrogen generation via NaBH_4_ hydrolysis.

**Supplementary Information:**

The online version contains supplementary material available at 10.1186/s13065-025-01646-4.

## Introduction

Rising global temperatures are caused by greenhouse gas emissions [1]. To meet the world's energy demands and encourage sustainable growth, alternative green energy sources must be discovered [2]. Hydrogen (H_2_) is a promising renewable energy carrier due to its unique advantages, such as being a clean energy resource [3], eco-friendly, with low emissions of greenhouse gases, high energy conversion performance, and superior energy density [4]. It has the highest energy value among energy sources, and is also the most plentiful element on Earth [5]. Hydrogen is categorized as blue, gray, brown, black, and green based on its source, production method, and environmental impact [6]. Blue hydrogen is generated from natural gas through steam methane reforming (SMR) [7, 8]. Gray hydrogen is produced through steam reforming or auto-thermal reforming of non-renewable fossil fuels, including coal or natural gas [9]. Nowadays, brown hydrogen, which is produced from environments rich in hydrocarbons, is the most abundant type, while coal gasification produces black hydrogen. Tons of CO_2_ are released into the atmosphere by each of these hydrogen production processes, or it is collected and stored underground [10]. On the other hand, green hydrogen is generated from renewable energy sources such as water and electricity through electrolysis or the hydrolysis of metal borohydride, with zero carbon emissions [11]. Different processes, including photocatalysis, catalytic hydrolysis of chemical hydrides, and seawater electrolysis, can be utilized to generate green hydrogen gas [12]. Despite seawater being recognized for its conductivity and abundance of ions, making it an excellent electrolyte for electrolysis while reducing costs, its high purity and corrosive ion content present a challenge for long-term electrode usage [13–17]. This leads to sluggish cathodic and anodic reactions [18, 19]. Therefore, the development of electrodes with both high activity and corrosion resistance is crucial for seawater electrolysis. Recently, scientists have been working on designing electrodes that are effective and stable specifically for seawater electrolysis [20, 21]. Chemical hydrides provide environmentally friendly hydrogen storage methods, such as metal boron hydrides and ammonia borane, which can meet the needs of industrial applications [22]. Sodium borohydride, a hydrogen-rich substance, is one of these sources and offers several advantages, including an effective and inexpensive method for generating hydrogen, its dehydrogenation and re-hydrogenation can be performed under mild conditions, a high level of safety during the storage process, and non-flammability [23, 24], excellent hydrolysis controllability, and a high degree of hydrogen purity. NaBH_4_ spontaneously hydrolyzes under ambient conditions according to the following Eq. (1) [25].

1 $$NaB{H}_{4}+\left(x+2\right) {H}_{2}O \to NaB{O}_{2}+x{H}_{2}O+4{H}_{2}$$

Although NaBH_4_ hydrolysis occurs naturally without a catalyst, an efficient catalyst is essential to maximize effectiveness and accelerate hydrogen release [26]. Thus, it is essential to utilize metal nanoparticles and nanocomposites as catalysts. Despite noble metal catalysts and metal–organic frameworks being demonstrated to be effective in promoting the hydrolysis of NaBH_4_, their high prices and scarce reserves prevent their widespread use [27].

Therefore, we focus on nickel oxide in our study as an alternate catalyst and non-precious material because of its unique activity in improving NaBH_4_ hydrolysis and facilitating its extensive application. Nickel oxide nanoparticles were synthesized using hydrothermal methods, calcined in the presence of oxygen, and then characterized using various techniques. FT-IR and XRD were used to investigate the presence of the nickel oxide functional group and the structural identification, respectively. SEM and TEM were used to examine the size and morphology of the synthesized nickel oxide particles. N_2_-adsorption was used to investigate the texture properties. Through XPS, several distinct surface species and their proportions were identified. In addition, we investigated how the concentration of NaBH_4_, solution temperature, and catalyst amount affected the hydrogen production rate of NaBH_4_ in the presence of the produced nickel oxide catalyst. Also, the activation energy was determined.

## Materials and methods

### Material

The following materials were purchased from Sigma Aldrich: 2-hydroxyethyl cellulose (HEC), sodium borohydride (NaBH_4_, 99%), nickel nitrate hexahydrate (Ni(NO_3_)_2_.6H_2_O), and aqueous Tetramethylammonium hydroxide (TMAH). Without purification, all reagents were utilized exactly as they were. Distilled water was utilized during the procedures.

### Catalyst preparation

**Scheme 1:**
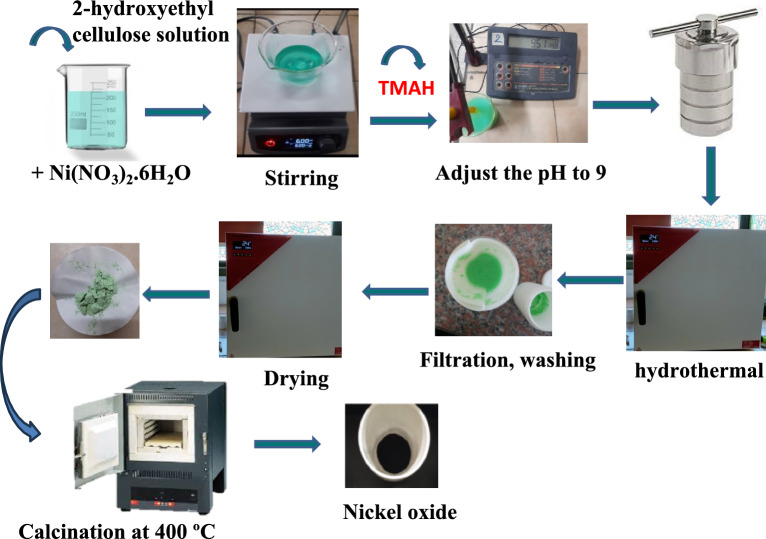
Representation of the steps performed during the synthesis of nickel oxide using the different templates by the hydrothermal technique

Hydrothermal synthesis was used to synthesize the nickel oxide. At different temperatures, 2-hydroxyethyl cellulose was used as the template and nickel nitrate hexahydrate as the precursor. In 100 ml of distilled water, a solution containing 0.021 M of nickel nitrate hexahydrate was prepared. A second solution of HEC was prepared by dissolving 1 g of it in 100 ml of distilled water at 60 °C until completely dissolved. The two solutions were then mixed in a 250 ml Teflon line, and the pH was adjusted to 9 using TMAH to ensure that a suitable base medium would be available for the precipitation of nickel hydroxide. The Teflon line was then placed in the autoclave and left at 100, 150, and 200 °C for 48 h. The precipitate was filtered and washed well with ethanol and distilled water several times, then placed for a whole night at 110 °C in a drying oven. The three samples calcined at a temperature of 400 °C with a rate of 1 °C/min for three hours isothermal (Scheme 1), the samples were noted as NH-100, NH-150, and NH-200, respectively.

### Materials characterization

The XRD pattern of NiO samples was obtained using D8-advance XRD and monochromatic Cu-Ka radiation (k = 1.5418 Å) at a scan rate of 0.05°/min in the 2θ range from 20° to 80°. The Bruker Alpha FT-IR instrument was carried out using Fourier-transform infrared (FT-IR) spectroscopy in the 400–4000 cm^−1^ range using KBr to investigate chemical functional groups. A transmission electron microscope (JEOL; model JEM 100 CX 11) and a SEM (JEOL JSM-5400 LV) operating at 15 kV were used to investigate the morphology of the different catalysts. TEM examination was performed at 200 kV in which the dried powder was dispersed in ethanol and sonicated for 15 min, 5 µL of the dispersed solution was placed on a 200-mish carbon-coated copper grid and then lifted until complete drying at room temperature. The size of the particles was measured using Gatan digital micrograph software, TEM model, JEOL, JEM-2100F, Japan. Brunauer–Emmett–Teller (BET) specific surface areas of the samples were obtained using a Belsorp-mini II (BEL JAPAN) nitrogen adsorption instrument at 77 K. The powders were degassed before surface area analysis at 200 °C for 6 h. Thermo VG Scientific spectrometer was used for performing the XPS measurement using an Al-Kα source (conditions: 13.5 kV, and 5 × 10^–10^ bar). The carbon 1 s peak appearing at binding energy (BE) of 284.6 eV was used as a reference.

### Catalytic experiments

A closed-glass apparatus, as reported by Dereń et al., was used for calculating the hydrogen evolved from the hydrolysis of NaBH_4_ [28]. The apparatus consists of two burettes (50 ml and 100 ml) filled with distilled water and a 150 ml tightly sealed round-bottom flask that was placed in a water bath and connected to a manometer. The ambient temperature of a 1-L beaker was adjusted before the reaction flask was placed inside. To calculate the amount of hydrogen evolving, the volume of water displaced by hydrogen was measured as a function of time. 10 mg of nickel oxide catalyst was thermostated at the appropriate temperature before each run. Subsequently, the catalyst vessel was instantly filled with 20 ml of 1.5 wt.% NaBH_4_, which had been thermostated at the same temperature, and connected to the glass apparatus. The volume of the generated hydrogen was calculated with the reaction time. A variety of variables, like reaction temperature (35, 40, 45, and 50 °C), catalyst amount (25, 50, 75, and 100 mg), and NaBH_4_ concentration (0.5, 1.5, 2.5, 3.5, and 4.5 wt.%), were studied for their effects on hydrogen production. The hydrogen yield, which is calculated by Eq. (2) and is the ratio of the total volume of hydrogen generated to the theoretical maximum volume of hydrogen, was used to measure the efficacy of hydrogen generation.

2 $$Yield \left(\text{\%}\right)=\frac{Total \,generated \,{H}_{2} \,volume}{Theoretical \,{H}_{2} \,volume}\times 100$$

Based on reaction stoichiometry and the assumption of ideal gas behavior under standard conditions (273 K, 1 atm), the theoretical hydrogen volume was calculated [29, 30].

3 $$L={n}_{{H}_{2}}\times \frac{22.4 L}{1 mol} \times \frac{absolute \,reaction \,temperature}{absolute \,standard \,temperature}$$

In Eq. (3), ${n}_{{H}_{2}}$ represents the moles of hydrogen determined by the stoichiometry of the reaction. Also, the rate of hydrogen generation (HGR) was defined as the volume of hydrogen produced per minute per gram of catalyst (Eq. (4)).

4 $$HGR=\frac{hydrogen \,volume (L)}{weight \,of \,catalyst \left(g\right)\times time (min)}$$

Equation (5) was used to calculate the activation energy using the logarithmic form of the Arrhenius equation.

5 $$lnk=lnA- \frac{{E}_{a}}{RT}$$

where A is the Arrhenius constant, k is the reaction rate constant, T is the reaction temperature, R is the universal gas constant (8.314 J/mol K), and E_a_ is the activation energy determined by J/mol. The recycling studies were used to examine the stability of the synthesized catalyst. After the first cycle of activity measurement, the catalyst was separated from the reaction vessel using a filter paper. Distilled water was used to completely wash the synthesized catalyst. After three hours of drying at 110 °C, and then reused for the next cycle of the catalytic measurements. Similarly, the catalyst's stability was investigated by repeating the experiment many times.

## Results and discussion

### Characterization of NiO NPs

**Fig. 1:**
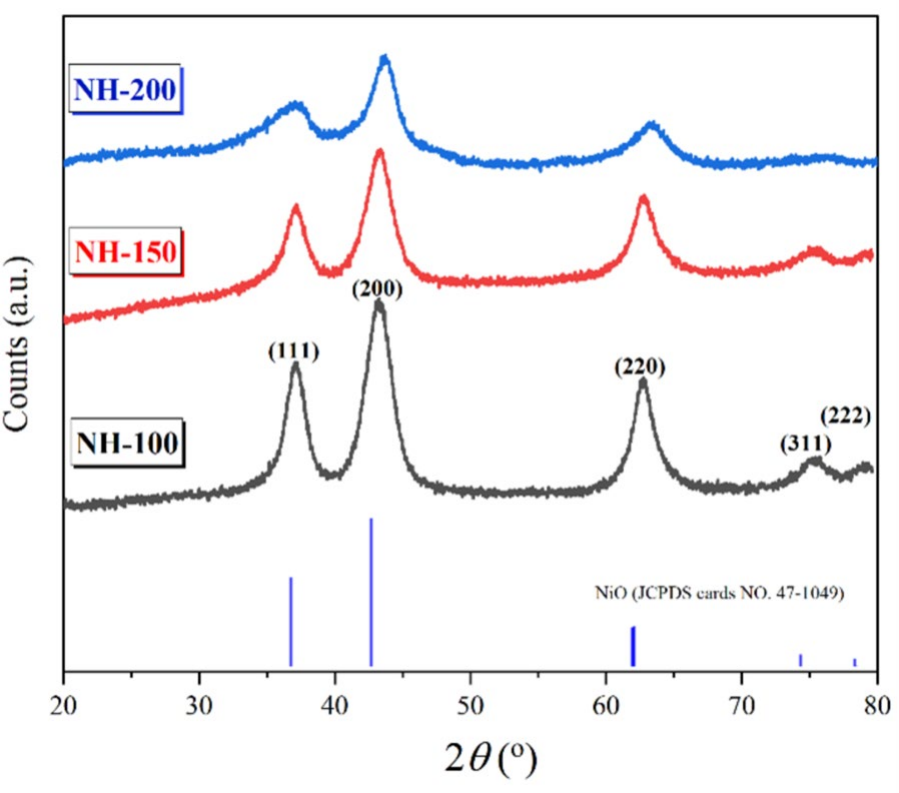
XRD patterns of NH-200, NH-150, and NH-100 catalysts

Fig. S1 illustrates the Ni(OH)_2_ XRD diffractogram, and the XRD data obtained were a good match with JCPDS no. 14–0117. Ni(OH)_2_ was then calcined at 400 °C to produce NiO. The XRD pattern of the NiO is illustrated in Fig. 1, which shows (111), (200), (220), (311), and (222) diffraction planes. Ni(OH)_2_ was successfully converted into NiO, as evidenced by the NiO XRD data matching the JCPDS card number 47-1049. Additionally, the broadened peaks in Fig. 1 indicate the nanometer-sized crystallites. Using the Debye–Scherrer equation, the crystal size was determined as follows:

6 $$ d{ } = \frac{{{\text{K}}\lambda }}{{{\text{B}}\cos \theta }} $$

where θ is the peak's diffraction angle, λ is the X-ray wavelength (Cu-Ka, 1.54056 Å), and B attitudes are the full width at half-height of the peaks (in radians). Grain size is represented by d. The Scherrer constant is K = 0.89. Using the Debye–Scherrer equation, the crystal size of the NiO sample was determined to be around 21 nm, 19 nm, and 13 nm for NH-100, NH-150, and NH-200, respectively. According to Bragg’s law, nλ = 2d sinθ, where d is the interplanar spacing. When Ni^3+^ ions (which have a smaller ionic radius than Ni^2+^) are present, the average Ni–O bond length decreases, leading to a reduction in the lattice spacing (d). Consequently, to satisfy Bragg’s condition, the diffraction angle (θ) must increase, resulting in a shift of XRD peaks toward higher 2θ values. Such peak shifts were observed in our XRD data (sample NH-200), indicating lattice contraction. This finding supports the assumption of an increased Ni^3+^ content and oxygen excess [31].

**Fig. 2:**
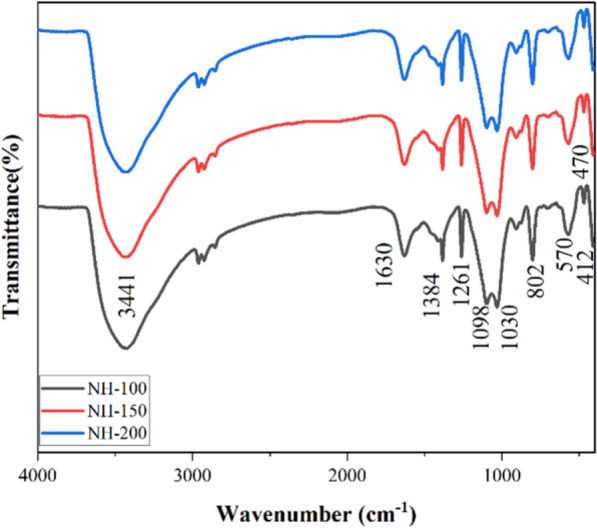
FT-IR spectra of NH-200, NH-150, and NH-100 catalyst

The FT-IR spectra between 400 and 4000 cm^−1^ are shown in Fig. 2. The broad absorption band indicates that the NiO particles are nanocrystals, while the peaks at 400 and 800 cm^−1^ can be attributed to the Ni–O stretching vibration mode. When compared to the bulk form, the FT-IR absorption of NiO nanoparticles is blue-shifted due to their spherical nanostructures and the quantum size effect [32, 33]. Ni–O–Ni stretching mode is responsible for the band at 1030 cm^−1^ and 1100 cm^−1^ [34, 35]. The vibrations of the carbonate ions may be the cause of the two sharp peaks seen at 1384 cm^−1^ and 1261 cm^−1^ [36, 37], or it might be identified as the C-H bond vibration's rocking mode as a result of the cellulose template [34, 35]. The O–H stretching vibration of the interlayer water molecules is responsible for the broad and intense band centered at 3441 cm^−1^, while the H–O–H bending vibration mode is assigned to the band near 1630 cm^−1^. These bands were also presented because the calcined powders have a tendency to physically absorb water or could be the result of water adsorption in the air during the preparation of FT-IR sample disks [33, 38, 39].

**Fig. 3:**
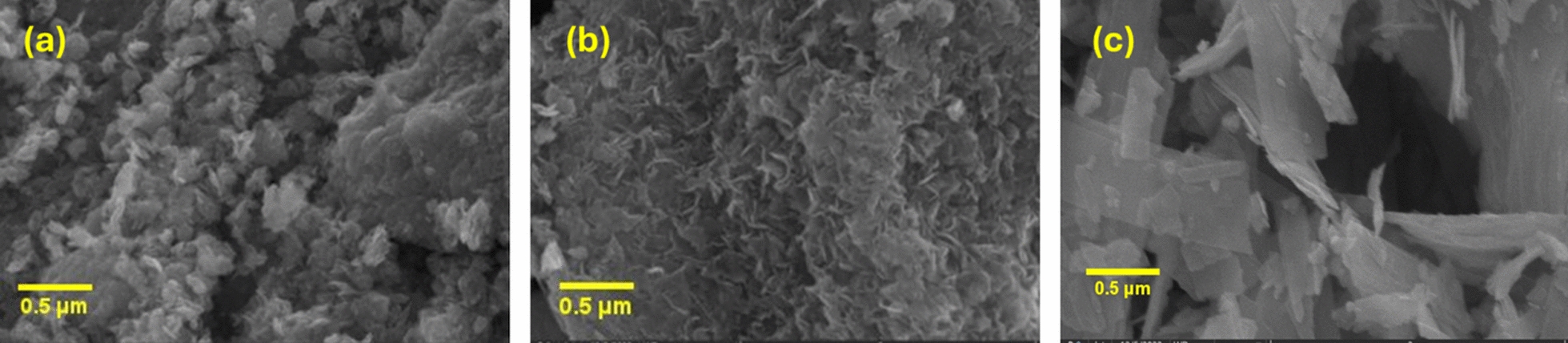
SEM images of **a** NH-100, **b** NH-150, and **c** NH-200 catalyst

**Fig. 4:**
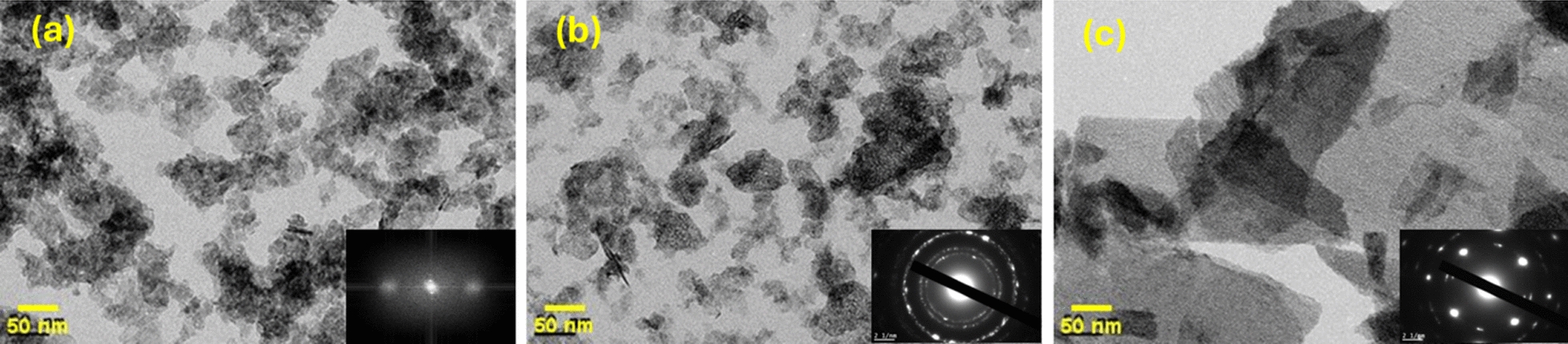
TEM images of **a** NH-100, **b** NH-150, and **c** NH-200 catalyst

To evaluate the effect of hydrothermal temperature on the morphology, the size and shape of the synthesized nickel oxide particles were characterized using scanning electron microscopy (SEM) and transmission electron microscopy (TEM). SEM images of NH-100, NH-150, and NH-200 are shown in Fig. 3. It is evident that the three catalysts possess varied morphologies. NH-100 possesses a morphology consisting of agglomerates with irregular shapes and randomly distributed pores among them, but NH-150 exhibits three-dimensional nanoflower morphologies, consisting of sheets. On the other hand, NH-200 revealed that the surface is nanosheets without any voids. The SEM images confirm the information presented in the TEM images of the hydrothermally synthesized nickel oxide. Furthermore, the highly crystalline character of the produced NiO NPs was shown by SAED [inset Fig. 4]. In the patterns, brilliant rings corresponding to the crystal planes (111), (200), (220), (311), and (222) are seen [40].

**Fig. 5:**
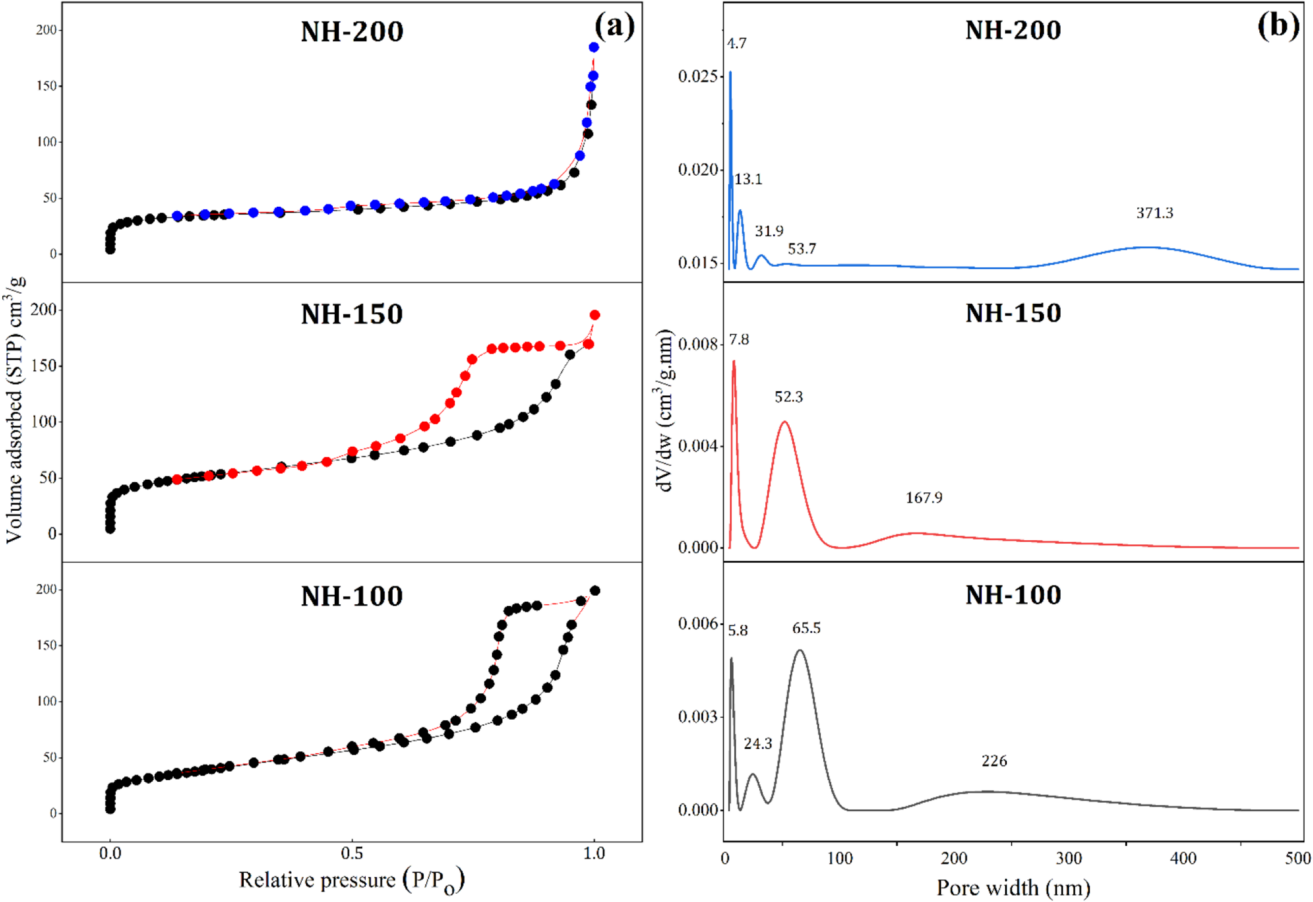
**a** N_2_ adsorption–desorption isotherms and **b** pore size distribution of NH-200, NH-150, and NH-100 catalysts

The surface area and porosity were measured using nitrogen adsorption–desorption isotherms. The pore size distribution curves and the resulting isotherms are shown in Fig. 5, while the specific textural parameters are listed in Table 1, which play a key role in the catalytic performance by influencing the accessibility of the active sites and the diffusion of the reactants. The N_2_ adsorption–desorption analysis revealed significant differences in the textural properties of the catalysts. Although NH-200 exhibited the lowest BET surface area (122 m^2^/g), the lowest external surface area (St = 50.7 m^2^/g), and the smallest pore volume (0.181 cm^3^/g), it demonstrated the highest catalytic activity in the hydrolysis of sodium borohydride. This superior performance is mainly attributed to its high microporous surface area (72.3 m^2^/g) and small crystallite size (13 nm). The high microporosity indicates many confined active sites, which enhances the adsorption and activation of NaBH_4_ and water molecules. In addition, the nanocrystalline nature provides more defect sites and enhances the surface reactivity. Interestingly, despite having the lowest external surface area, NH-200 still outperformed the others. This suggests that in this particular reaction system, the internal microporous surface plays a more dominant role than the external surface area. This implies that the reaction mechanism may favor confined spaces that help orient or stabilize the intermediates. In contrast, NH-150, which had the highest BET and external surface area, exhibited lower activity, possibly due to a less favorable pore structure or a lower concentration of truly active sites. Thus, the catalytic efficiency is governed by a combination of the microporous structure, crystallite size, and the chemical nature of the active sites, rather than the surface area alone.

**Table 1:** Textural characteristics of the synthesized nickel oxide

Catalyst	Crystallite size (nm)	Surface area (m^2^·g^−1^)	S_t_	S_mic_	V_p_/cm^3^g^−1^
NH-100	21	138.9	130.5	8.4	0.296
NH-150	19	181.8	134.4	47.5	0.263
NH-200	13	122.9	50.7	72.3	0.181

According to the IUPAC classification [41, 42]. The obtained isotherms indicate type IV behavior, meaning the presence of mesopores in all synthesized catalysts. Above a relative pressure value of 0.7, there is a notable and rapid rise in the adsorbed volume, indicating two important observations. First, it indicates that nitrogen (N_2_) undergoes capillary condensation within the mesopores of the catalyst. Second, it indicates that the synthesized catalysts exhibit a uniform distribution of pore sizes. Additionally, the desorption of N_2_ molecules from the adsorbents was determined to be irreversible, as evidenced by the presence of hysteresis loops in the data. These hysteresis loops fall under the H1-type classification according to the IUPAC system [43].

**Fig. 6:**
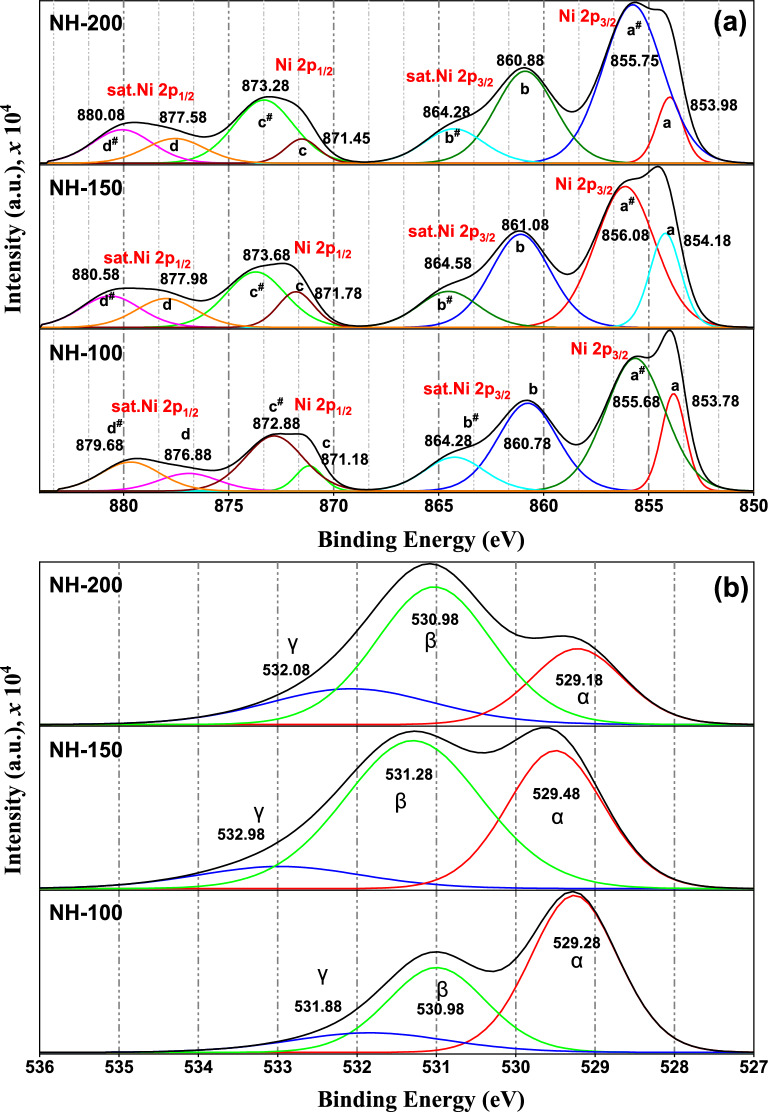
XPS results of the synthesized NiO: **a** Ni 2p fitted spectrum, **b** O 1 s fitted spectrum for NH-200, NH-150, and NH-100 catalyst

The stoichiometry and chemical characteristics of the catalysts were examined using XPS analysis, which may also provide information on the oxidation state of active Ni species in the upper layers of the catalysts' surfaces. Figure 6 indicates the high-resolution XPS spectra of Ni (2p) core level obtained for the synthesized NiO samples. The corresponding results from the details spectra indicate that Ni and O existed in the prepared samples, which include Ni^2+^ and Ni^3+^ (Table 2). The structure of the synthesized samples varies due to the presence of excess oxygen in the samples. Because the radius of Ni^3+^ ions is smaller than that of Ni^2+^ ions, the Ni^3+^–O^2−^ bond in samples with higher oxygen contents contributes to the shorter bond distance [44].

**Table 2:** Ni^3+^/Ni^2+^ ratio and atomic percentages of the elements detected in synthesized nickel oxide

	NH-100	NH-150	NH-200
Ni^3+^/Ni^2+^ ratio	3.35	2.92	5.59
Ni^2+^(2p_3/2_) binding energy	853.78	854.18	853.98
Ni^2+^(2p_1/2_) binding energy	871.18	871.78	871.45
Ni^3+^(2p_3/2_) binding energy	855.68	856.08	855.75
Ni^3+^(2p_1/2_) binding energy	872.88	873.68	873.28
O 1* s* (at. Percent)	56.2	63.1	69.3
Ni 2*p3*(at. Percent)	43.8	36.9	30.7
O/Ni ratio	1.28	1.70	2.26

It was observed from (Fig. 6) that the Ni 2p peak could be split into eight peaks -No peaks of other elements are observed- which appeared over all the synthesized catalysts located at various binding energies which were marked as a, a^#^, b, b^#^, c, c^#^, d, and d^#^, which are referred to Ni^2+^(2p_3/2_), Ni^3+^(2p_3/2_), Ni^2+^(2p_3/2_) (satellite), Ni^3+^(2p_3/2_) (satellite), Ni^2+^(2p_1/2_), Ni^3+^(2p_1/2_), Ni^2+^(2p_1/2_) (satellite), and Ni^3+^(2p_1/2_) (satellite), respectively [45–48]. The peaks at 855.3 and 873.3 eV were attributed to the presence of Ni^3+^ species. These two peaks are separated by 17.7 eV. Four more satellite peaks are located with binding energies of 860.8, 864.2, 878.9, and 879.9 eV, in addition to the Ni(2*p*) peaks [47, 48].

The O 1 s spectra were fitted using a three-component model to account for the various oxygen environments typically present in NiO-based nanomaterials. The first peak (O_α_), located around 529.18–529.48 eV, corresponds to lattice oxygen (O^2−^) in NiO. The second component, centered at approximately 530.98–531.28 eV, is assigned to surface-adsorbed oxygen species (O_β_), including hydroxyl groups (–OH) or chemisorbed O^−^. The third broader feature (O_γ_) around 531.88–532.08 eV is attributed to molecularly adsorbed water. Regarding the correlation between the O 1 s and Ni 2p signals, a consistent trend is observed: samples with higher intensity in the O_β_ component of the O 1 s region (notably NH-200) also exhibit more prominent Ni^3+^ features and satellite structures in the Ni 2p spectra. This may suggest an increased degree of surface hydroxylation, lattice defects, or non-stoichiometry [49, 50].

The XPS spectra clearly show variations in the binding energies (BE) of the Ni 2p3/2 peaks at 853.98 eV, 854.18 eV, and 853.78 eV for NH-200, NH-150, and NH-100, respectively. These shifts are significant and should be discussed in terms of the chemical environment and electronic interactions of Ni species within each sample. Although NH-200 shows a slightly higher O_β_/Ni^3+^ ratio (0.55) compared to NH-150 (0.48) and NH-100 (0.22), a noticeable shift toward higher binding energies was observed in the NH-150 sample. This observation suggests that BE is not governed solely by the quantity of surface oxygen, but also by the chemical nature of the species involved, the degree of hydroxylation, and local structural features. NH‑150 may contain a higher fraction of strongly bound hydroxyl groups (Ni-OH) or chemisorbed O^−^ species, which create a more pronounced local positive charge on Ni atoms, producing a larger BE shift, even with slightly lower O_β_ content. Additionally, NH‑150 might have more surface defects or lattice distortions that enhance charge localization and increase BE. In contrast, NH-200, despite its higher O_β_/Ni^3+^ ratio, may exhibit a more crystalline structure or stronger Ni^3+^ screening effects that mitigate the BE shift. These effects are consistent with Biesinger et al. [51] and Natascha Weidler et al. [52], who emphasized that BE values reflect both oxidation states, the chemical and structural environment of the element.

### NaBH_4_ hydrolysis activity

**Fig. 7:**
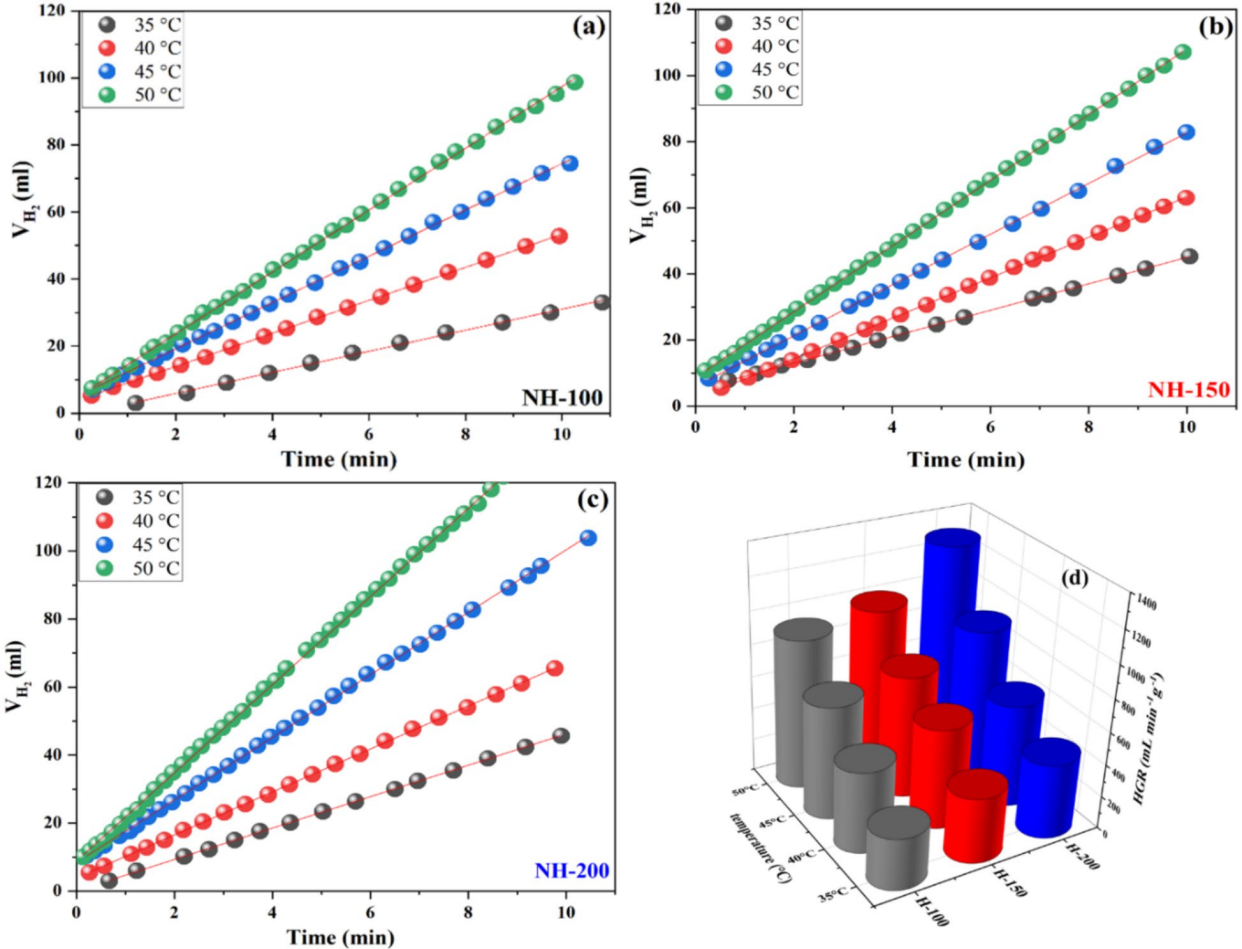
The effect of catalytic reaction temperature on **a**, **b**, and **c** evolved hydrogen, **d** HGR for NH-200, NH-150, and NH-100 catalyst

**Scheme 2:**
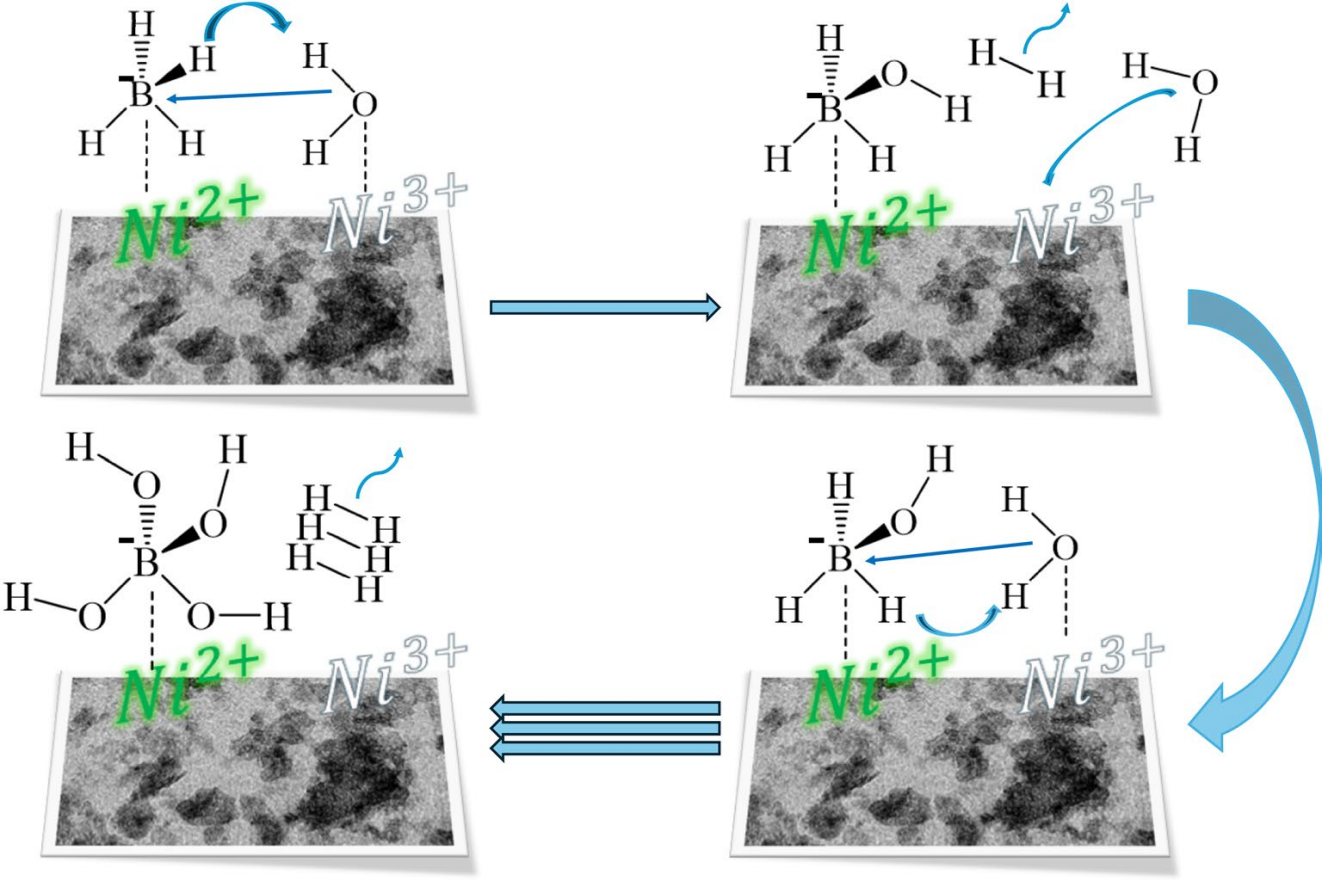
Schematic representation of Langmuir–Hinshelwood mechanism for the hydrolysis of sodium borohydride catalyzed by nickel oxide nanoparticles

The efficiency of synthesized nickel oxide as a catalyst to produce hydrogen via NaBH_4_ hydrolysis was investigated. Using Eq. (4) and the information presented in Fig. 7, the rate of hydrogen production (HGR) was calculated. After a longer period of NaBH_4_ hydrolysis, a noticeable color change of the catalyst from black to greenish gray was observed, which may indicate a partial reduction of surface Ni^3+^ species to Ni^2+^. Although post-reaction XPS characterization could not be performed due to rapid oxidation of the catalyst upon exposure to air, this observation suggests that Ni^3+^ sites might undergo reduction during the catalytic process, which reduced the reaction rate (Fig. S2), and that illustrates the significant role of Ni^3+^ ions in the rate of hydrogen production (Table 3). Scheme 2 represents the catalytic hydrolysis of sodium borohydride over nickel-based catalysts can be interpreted via a Langmuir–Hinshelwood–type mechanism, in which both BH_4_^−^ and H_2_O molecules are adsorbed and activated on distinct active sites [53]. Specifically, Ni^2+^ ions act as electron-rich centers that facilitate the adsorption and activation of BH_4_^−^ species due to their ability to donate electron density to the boron atom. This electron transfer weakens the B–H bonds and promotes the formation of reactive intermediates such as BH_3_(OH)^−^. Conversely, Ni^3+^ sites behave as electron-deficient centers with strong Lewis acidity, enhancing the adsorption and polarization of H_2_O molecules. The interaction with Ni^3+^ pulls electron density from the oxygen atom in water, making the proton more electrophilic and thus more susceptible to attack by the hydride ion from BH_4_^−^. The reaction between H^−^ (from BH_4_^−^) and H+ (from water) leads to the evolution of molecular hydrogen (H_2_). The balance between Ni^2+^ and Ni^3+^ species plays a critical role in determining the catalytic activity. A higher Ni^2+^/Ni^3+^ ratio typically favors BH_4_^−^ activation, while sufficient Ni^3+^ concentration ensures efficient H_2_O activation. However, an optimized ratio is necessary to achieve synergy between both sites, ensuring a high turnover frequency and H_2_ generation rate. Therefore, tuning the oxidation state distribution within the catalyst surface is a key strategy to enhance performance.

**Table 3:** The relation between the increased Ni^3+^ content and the improvement in catalytic performance

Sample	HGR at 323 K (ml/min/g)	Ni^3+^/Ni^2+^ ratio	Activation energy (KJ/mol)
NH100-C	918	3.35	59.2
NH150-C	990	2.92	48.9
NH 200-C	1290	5.59	57.8

We used the Arrhenius equation to obtain the activation energy (E_a_) of the catalytic process. Four reaction temperatures were selected to show the effect of the reaction conditions on the hydrogen production rate. The values of E_a_ for NH-100, NH-150, and NH-200 were found to be 59.2, 48.9, and 57.8 kJ/mol, respectively, by plotting the natural logarithm of HGR (ln HGR) versus the reciprocal of the absolute temperature (1/T). Table 4 presents a comparison of our catalyst performance with other reported nickel-based or non-precious metal catalysts. Data indicate that an optimal Ni^3+^/Ni^2+^ ratio plays a crucial role in lowering the apparent activation energy. For instance, among the studied samples, NH150-C showed the lowest E_a_ value, which correlates with its moderate Ni^3+^/Ni^2+^ ratio. Hence, the combination of both species at a balanced ratio helps lower the energy barrier for the reaction. Furthermore, it is important to highlight that E_a_ is governed by multiple factors, not only the oxidation state distribution. In particular, the higher surface area (181.8 m^2^.g^−1^) and pore volume observed for NH150-C, along with its excess surface oxygen defects, may have also contributed to lowering the energy barrier. Therefore, the enhanced activity of NH150-C may be due to the result of a synergistic combination of structural, textural, and surface chemical properties, rather than a single dominant factor.

**Table 4:** Hydrolysis of sodium borohydride using different catalysts

Catalyst	Conditions	HGR, ml·g^−1^·min^−1^	Activation energy (KJ/mol)	References
Ni-based thin film catalyst	1.15 g cat, 3.8% NaBH_4_, 35°C, pH 5	680	91.7	[54]
Ni NPs catalyst synthesized via thermal plasma	0.02 g cat, 0.793 M NaBH_4_, 27°C, pH 9	171	69.7	[55]
NiB/NiFe_2_O_4_	0.1 g cat., 0.5 wt% NaBH_4_, 25°C	299.9	72.5	[56]
NiFe_2_O_4_	0.01 g cat., 0.66 wt% NaBH_4_, 70°C	2181	50	[57]
Ni–Co–B	2.7 wt.% or 0.16 g of 5 ml of NaBH_4_ solution, 28 °C	2608	64.8	[58]
NH100-C	0.01 g cat, 1.5% NaBH_4_, 35 °C, pH 10	310	59.2	This work
NH150-C	0.01 g cat, 1.5% NaBH_4_, 35 °C, pH 10	396	48.9	This work
NH200-C	0.01 g cat, 1.5% NaBH_4_, 35 °C, pH 10	456	57.8	This work

**Fig. 8:**
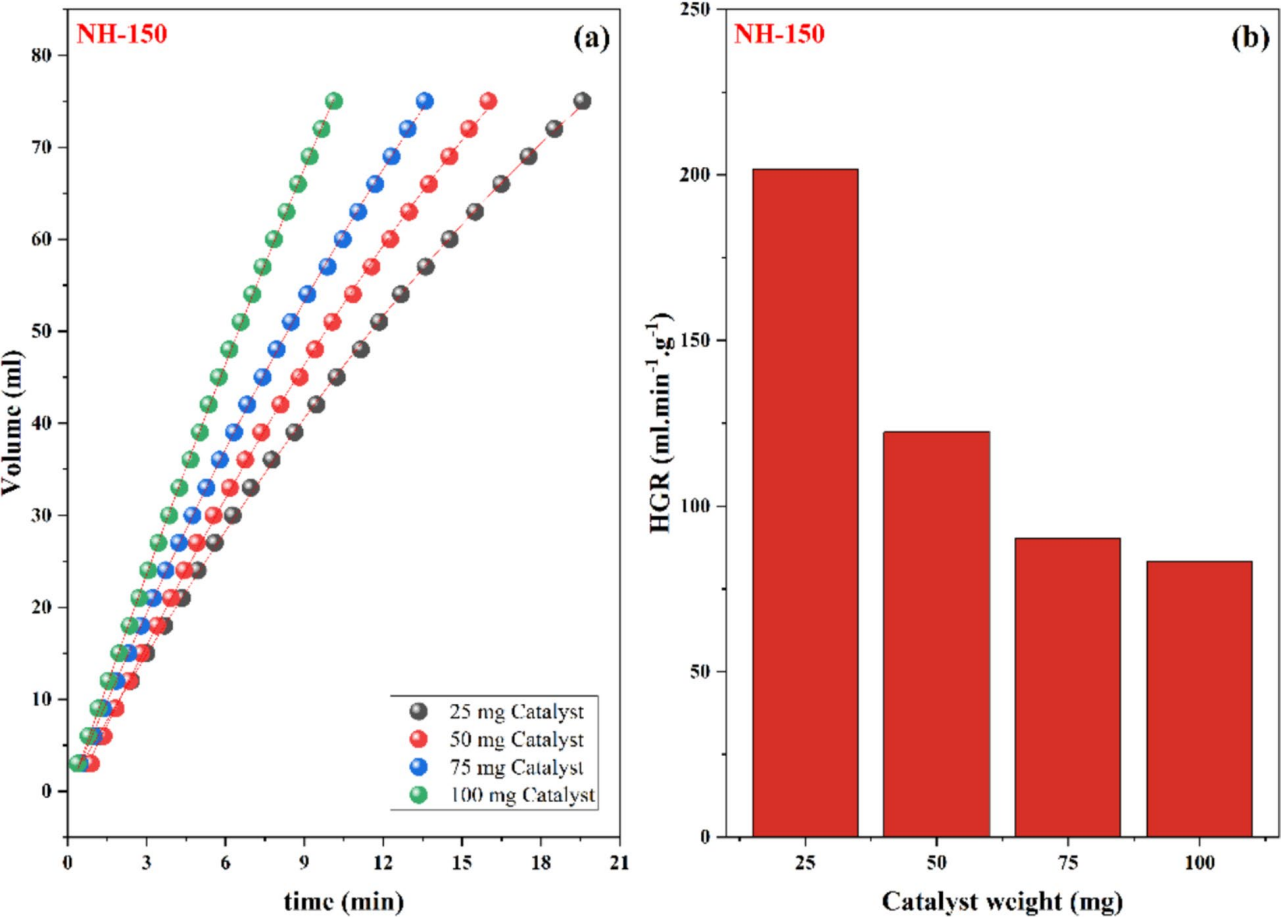
The effect of catalyst weight on **a** evolved hydrogen, **b** HGR for NH-150 catalyst. (conditions: NaBH_4_ 1.5 wt% and 40 °C)

**Fig. 9:**
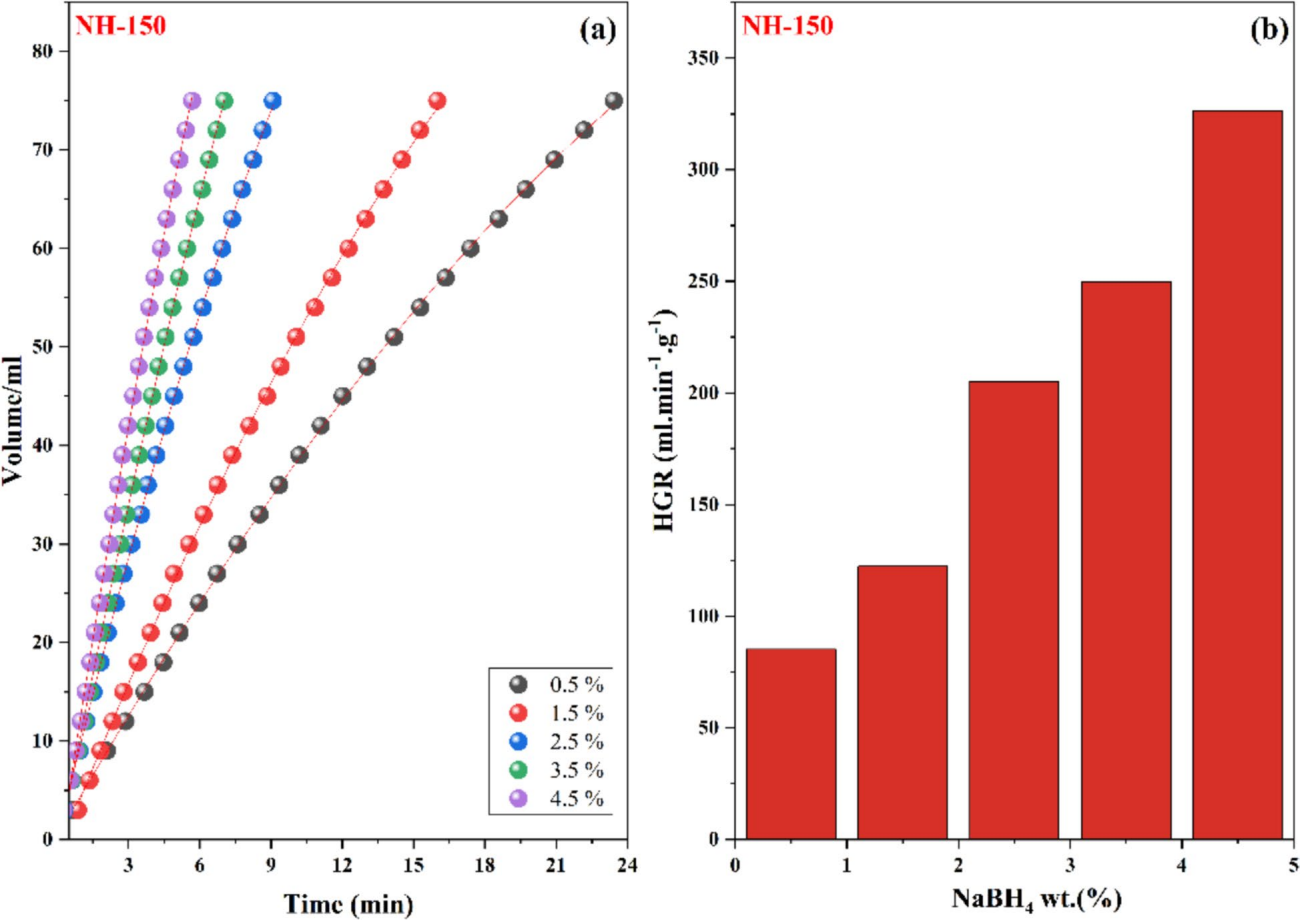
The effect of the concentration of sodium borohydride on **a** evolved hydrogen, **b** HGR for NH-150 catalyst (Conditions: 50 mg cat. and 40 °C)

To determine the catalyst's effect on hydrogen generation. The amount of synthesized nickel oxide (NPs), which was used as a catalyst, ranged from 25 to 100 mg, while other reaction parameters such as NaBH_4_ concentration (1.5 wt.%) and temperature (313 K) were maintained constant. As expected, increasing the amount of catalyst from 25 to 100 mg significantly reduced the time needed to produce a specific volume of hydrogen (Fig. 8). This is explained by the fact that the catalyst surface has a greater number of active sites with which the reactant molecules may interact. As the amount of catalyst increased, the hydrogen generation rate per gram (HGR) lowered from 200 to 83 mL/min/g, this resulted in a buildup of hydrogen gas and a decrease in the HGR at higher NaBH_4_ concentrations. The effect of NaBH_4_ concentration on the hydrogen generation performance of NH-150 was also investigated at fixed catalyst dosage (10 mg). As shown in Fig. 9, increasing the NaBH_4_ concentration from 0.5 to 4.5 wt.% significantly enhanced both the volume of evolved hydrogen and the hydrogen generation rate (HGR). This enhancement is likely attributed to the increased availability of BH_4_^−^ ions, which accelerates the hydrolysis reaction. However, it is important to note that beyond a certain concentration, the system may experience limitations such as local depletion of water or mass transfer effects, but no such decrease was observed under the tested conditions (Table 5).

**Table 5:** Effect of NaBH_4_ concentration on the HGR

Concentration of NaBH_4_ (wt.%)	Time (min)	Rate (mL/min/g)
0.5	5.7	85.2
1.5	7	122.4
2.5	9	205.2
3.5	16	249.6
4.5	23	326.4

**Fig. 10:**
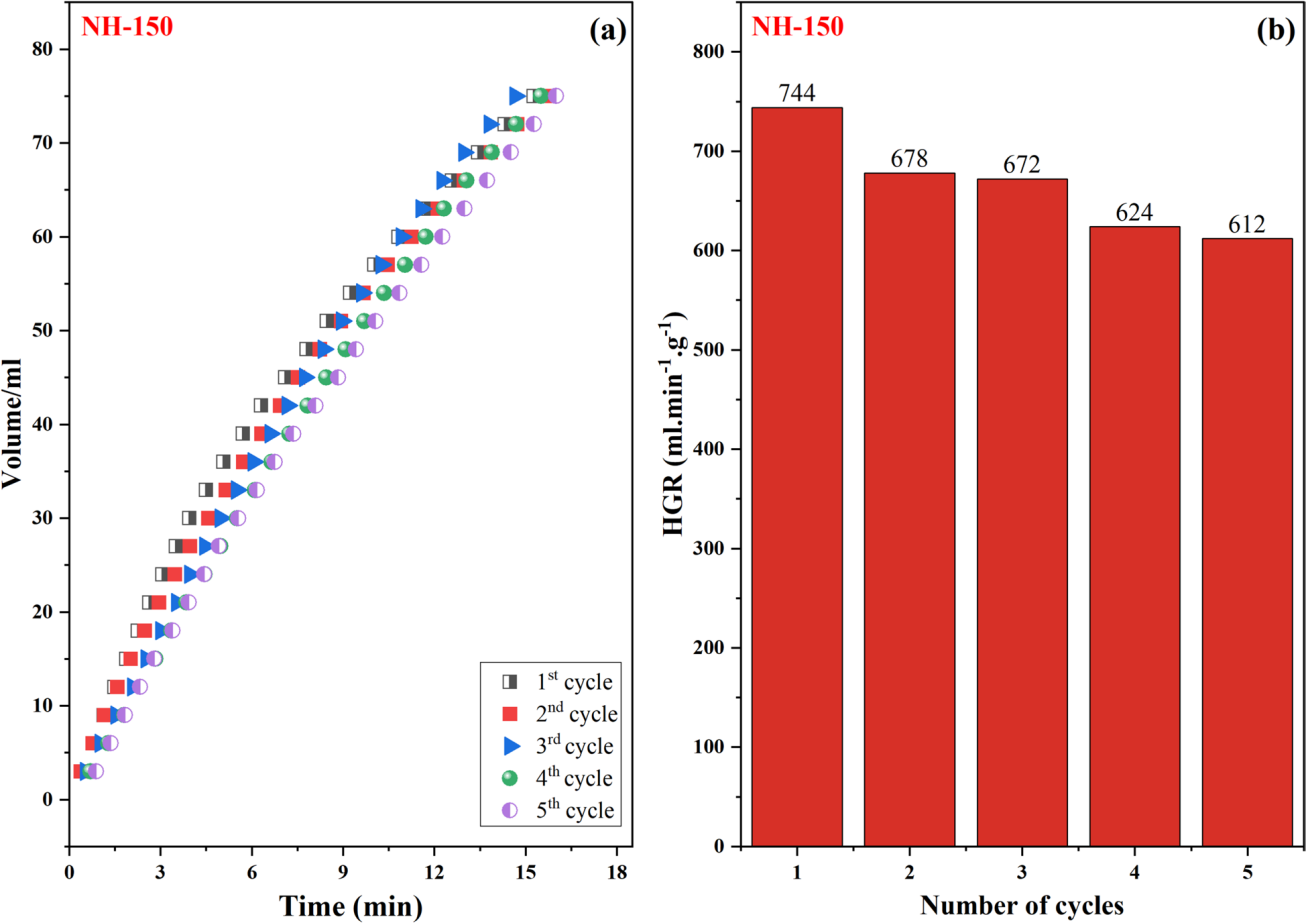
Variation of evolved hydrogen HGR over NH-150 catalyst for five cycles (Conditions: 50 mg, 40 °C, 1.5% NaBH_4_)

The effect of temperature on the rate of hydrogen production using NiO nanoparticles (NPs) for NaBH_4_ hydrolysis was studied at 308, 313, 318, and 323 K (Table 3). Even at a relatively low temperature of 308 K, the NiO NPs proved a noticeable activity for hydrogen production, generating hydrogen at a rate of 456 mL/min/g using NH-200 (likely a specific type of NiO NPs). This proves the exothermic nature of the NaBH_4_ hydrolysis (it releases heat) and provides some energy to accelerate the reaction even at lower temperatures. However, increasing the reaction temperature to 323 K significantly increased the HGR to 1290 mL/min/g while using NH-200 as a catalyst. This improvement can be attributed to the increased frequency of "effective collisions" between NaBH_4_ molecules, water molecules, and NiO NPs at higher temperatures [48, 49]. These encounters are more likely to result in a successful reaction, and hence a greater rate of hydrogen creation. In conclusion, the results clearly reveal a favorable association between the reaction temperature and the rate of hydrogen generation via NaBH_4_ hydrolysis with NiO NPs. The stability of the NiO NPs was examined by reusing the catalyst five times for the production of hydrogen at 40 °C and under constant operating conditions (50 mg catalyst, 1.5 wt.% NaBH_4_). At the conclusion of each cycle, the used catalyst was collected. The catalyst was washed many times with distilled water and ethanol to reach a neutral pH value. Finally, the washed catalyst was oven-dried at 120 °C for 12 h. Figure 10 shows that as the number of cycles increased, the catalyst activity decreased slightly. The active sites on the NiO surface may have been partially inhibited by surface contamination, mostly caused by the accumulation of byproducts (e.g., NaBO_2_).

## Conclusions

Our study shows a systematic association between the hydrothermal synthesis temperature, surface/microstructural characteristics, and the catalytic efficiency in NiO. Unlike prior studies, the synthesis conditions were varied systematically (NH-100, NH-150, NH-200), allowing us to adjust the textural and structural properties (such as crystallite size, surface area, and microporosity) and directly relate them to the catalytic activity. The NH-200 sample exhibited high catalytic activity, despite its low BET and external surface area, which is attributed to its microporous structure and nanoscale crystallite size, a factor that has previously been overlooked in NiO hydrolysis studies. Our research underscores the significance of the microporosity and internal surface area over the external surface area in this reaction system, offering new perspectives on designing more effective NiO-based catalysts. To the best of our knowledge, there are few studies that have methodically examined the textural characteristics of NiO in this manner and directly connected them to the performance of NaBH_4_ hydrolysis. On the other hand, this work established a direct relationship between the Ni^3+^/Ni^2+^ ratio (via XPS analysis) and the catalytic activity, which is not often quantified in similar works. Finally, it has been demonstrated that nanostructured NiO can be effectively tuned through a rapid, cost-effective, and industrially scalable hydrothermal process by precisely controlling the reaction temperature, without requiring modifications to other synthesis parameters.

## Supplementary Information

Additional file 1.

## Data Availability

No datasets were generated or analysed during the current study.

## Abbreviations

BE: Binding energy
BET: Brunauer–Emmett–Teller
FT-IR: Fourier-transform infrared spectroscopy
HGR: Hydrogen generation rate
JCPDS: Joint Committee on Powder Diffraction Standards
SAED: Selected area electron diffraction
SEM: Scanning electron microscopy
TEM: Transmission electron microscopy
XPS: X-ray photoelectron spectroscopy
XRD: X-ray diffraction
