# Supplementary material for: Nickel oxide nanoparticles catalyst for enhancing green hydrogen production: effect of preparation conditions
Source: BMC Chem. 2025 Oct 27;19(1):285. doi: 10.1186/s13065-025-01646-4 (PMC12560531; doi:10.1186/s13065-025-01646-4)
Supplement: Supplementary file 1 — Additional file 1. [file 13065_2025_1646_MOESM1_ESM.docx]

**Nickel Oxide Nanoparticles Catalyst for Enhancing Green Hydrogen Production: Effect of Preparation Conditions**

Hatem A. Mahmoud **^1^,** Aya Adel A. Ali **^1^,** Tarek T. Ali **^1^**, Bahaa M. Abu-Zied **^2^**

^1^Chemistry Department, Faculty of Science, Sohag University, Sohag 82524, Egypt

^2^Chemistry Department, Faculty of Science, Assiut University, 71516 Assiut, Egypt

**Figure S1- XRD patterns of NH-200, NH-150, and NH-100 before calcination.**

**Figure S2** **The effect of catalytic reaction temperature evolved hydrogen**
